# The Bending Impact of the Failure Investigation of the Polymer-Reinforced Composite Protection Bars

**DOI:** 10.3390/polym18081001

**Published:** 2026-04-21

**Authors:** Ibrahim Kutay Yilmazcoban

**Affiliations:** Department of Mechanical Engineering, Faculty of Engineering, Sakarya University, Sakarya 54187, Türkiye; kyilmaz@sakarya.edu.tr; Tel.: +90-264-295-57

**Keywords:** polymer-reinforced composite, tube, bending impact, energy absorption, explicit finite element method, fracture, bending failure

## Abstract

It is well established that an anti-intrusion beam is a passive safety system that serves an essential role for passengers during collisions. In this study, the influence of internal reinforcements on the bending failure of a cylindrical aluminum tube was systematically investigated through a series of composite beam tests. Polymeric materials, including cast polyamide (PA6) and polypropylene (PP), with varying wall thicknesses, were deemed suitable for use as the inner reinforcement of the Al 6063-T6 tube. The test setup, which simulates impact conditions experienced by structural components in full-scale crash tests, is a powerful tool for the bending impacts in the study. To describe the connection between bending impact and quasi-static loading of composite beams, each method is compared to clarify the composite’s failure behavior. An explicit Finite Element Analysis (FEA) of impact scenarios has been performed to understand the deformation behavior of polymer-reinforced composites and to determine the absorbed impact energy, thereby clarifying which specimen is better able to absorb bending impact energy. Primarily, three polymer-reinforced specimens were accepted with a hollow Al tube. After initial tests and simulations, the expected parametric study could not be achieved except for one. Then, three more combinations were offered. For one of the three specimens, the thickness of the central reinforcement PP was increased until a fully developed shaft was produced, resulting in better-than-expected bending impact-absorbing performance. The results indicate that the energy level of the inner reinforcements with polymeric materials increased 8.8 times, to about 750 J, compared to the plain Al tube (85 J) under bending impact loads. The numerical simulations are relevant and reliable for the details of the specimens’ impact process and show good agreement with the experimental results. Finally, depending on the content, this research, rather than focusing on the fundamental concept of polymer-reinforced aluminum crash tubes, focuses on the specific dynamic bending impact evaluation of the Al, PA6, and PP configuration and the design insight that hollow PP reinforcement can accelerate fracture. In contrast, a fully filled PP core inside a PA6 sleeve can suppress splitting and substantially improve impact energy absorption.

## 1. Introduction

The crash and impact protection bars are essential components of vehicles, protecting drivers and passengers from collisions. During impact, the collision energy is absorbed by the bars and beams. Recently, materials, including composites and aluminum alloys, have been used in various applications to absorb the impact energy. Bumper supporting structures have recently been made from aluminum extrusions rather than steel to reduce weight, as with many other parts. The protection mechanisms should be understood to develop crash bars, and the experiments establish the mechanical behavior of tubular structures. After tensile and compression tests, the bending test is the most common and beneficial test method. Various studies address the approximation of the bending behavior of aluminum tubes [1,2,3,4,5,6,7,8]. The bending collapse of thin-walled structures is localized in a plastic hinge mechanism, and the remaining zones of the beams undergo a rigid-body rotation. Due to a slight rotation angle, local collapse occurs in the beam, and its resistance drops significantly, resulting in a low energy absorption efficiency [9,10,11,12]. Kecman showed a comprehensive experimental and theoretical investigation of the bending performance of square and rectangular prismatic beams [13]. Kecman proposed simple failure mechanisms involving stationary and moving plastic hinge lines. Kecman’s theory clarifies that the material is sufficiently ductile not to fracture during collapse. This theory was also verified by the quasi-static bending tests. Abramowicz [14] independently developed a similar formula. McGregor et al. [15] reported the experimental results of bending the aluminum hat sections.

Since cellular structures and metal foams have the flair to absorb a large amount of energy when severely deformed, introducing lightweight metal fillers into thin-walled beams has attracted increasing interest [1,2,4,8]. Low-weight foams within beams reduce rebound after compression, enabling the use of thinner profiles and greater design freedom [2]. A pilot study on the bending collapse of thin-walled beams with a lightweight metal filler was delivered by Santosa and Wierzbicki [16]. Chen et al. developed an analytical formulation to determine the optimal honeycomb and foam-filled beams under bending load [17].

Some recent studies have focused on the bending performance of composite beams, considering both experimental and numerical simulation work on bending behavior under impact loading conditions, indicating the development of explicit Finite Element Method (FEM)-based approaches [17,18,19,20,21,22].

In this paper, the influence of inner polymer reinforcements on the impact energy absorption capacity of a cylindrical aluminum tube was systematically investigated through a series of reinforcement-combination simulations and experiments. However, the bending impact-absorbing performance/mass ratio was maximized by varying the thicknesses of the inner fillers. Bending impact tests and explicit FEM simulations were performed using ANSYS/LSDYNA to determine the appropriate combinations. The study focuses on improving the bending impact failure of a hollow Al tube using different inner polymeric fillers with varying thicknesses, using pendulum impact tests and explicit finite element simulations to develop a reliable polymer-reinforced passive protection bar. Primarily, inside the hollow Al tube, a 4 mm thick PA6 (cast polyamide) was considered as a first inner filler, and, later on, different thicknesses of PP (polypropylene) as a second filler, used for bending impact simulations to compare all experiments. After initial tests and simulations, the expected optimization could not be achieved. The thickness of the central reinforcement PP was increased until a fully developed shaft was produced, resulting in better-than-expected bending impact-absorbing performance. The final tubular composite optimization compared with the plain Al tube is, first, a 4 mm thick, 29 mm outer diameter PA6 shaft; second, a 21 mm outer diameter full PP shaft. Consequently, the plain hollow Al tube’s mean impact energy (85 J) absorption level increased by 8.8 times with the mean absorbed energy of the inner polymeric material-reinforced Al (750 J).

Eventually, depending on the content, this research, rather than focusing on the fundamental concept of polymer-reinforced aluminum crash tubes, focuses on the specific dynamic bending impact evaluation of the Al/PA6/PP configuration and the design procedures. While the hollow PP reinforcement can accelerate fracture, a fully filled PP core inside a PA6 sleeve can suppress splitting and substantially improve impact energy absorption.

Finally, the main idea behind the almost monolithic polymeric support inside the Al tube for the most effective specimen is its ease of availability in the market and manufacturability, as well as its economical choice for the industry, which is almost the most important factor given the safety standards.

## 2. Materials and Methods

### 2.1. Material Properties

The used aluminum tubes were made from commercial-quality extruded 6063-T6 aluminum supplied by Ada Metal, Sakarya, Türkiye. PA6 and PP were considered for the inner reinforcement materials, which the same company also provided. The reason for preferring PA6 is its superior intensity and stiffness, which support the hollow Al tube as an initial layer. As a second inner filler, PP is a reliable choice for absorbing shock and reducing its effects due to its toughness.

**Figure 1:**
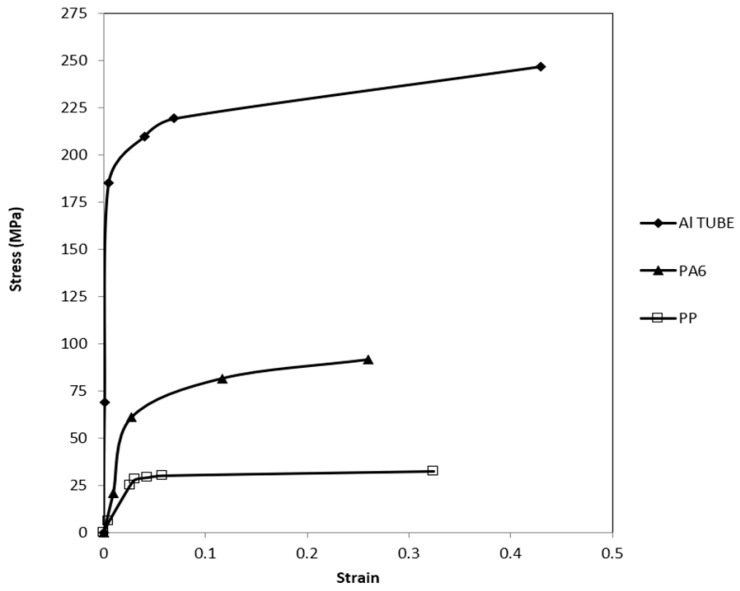
Tensile test results of the materials used for the specimens.

The mechanical properties of the aluminum tube and the polymeric materials were determined from tensile tests according to ASTM E8/E8M-13a [23] and ASTM D638-10 [24], respectively. A video extensometer-adapted MTS universal test setup was used to execute the quasi-static tests. The tensile test results for the materials used are shown in Figure 1.

The significant aspect of this study is the impact behavior; instead of using quasi-static bending simulations, the quasi-static test results were used. Therefore, the properties given in Table 1 are valid only for impact simulations with the ANSYS/LSDYNA 2024 R1 software.

To better illustrate the study, the physicochemical rationale for selecting the PA6-PP pair for reinforcing the aluminum tube is as follows.

PA6 is a polar, hydrogen-bonding polymer with a strong affinity for adhesives and metal surfaces. It can form stable interfacial interactions with aluminum, improving load transfer. PA6 has a relatively high melting point (~220 °C) and good thermal stability, making it suitable for structural reinforcement. PA6 absorbs water (up to ~3–4% by weight), which can reduce stiffness. It has high tensile strength (~80–90 MPa), good modulus (~2.25 GPa), and excellent toughness.

PP is non-polar, chemically inert, and resists moisture uptake. It acts as a stabilizing layer, reducing environmental sensitivity and creep. PP melts at a lower temperature (~165 °C), but its crystallinity and toughness provide impact resistance. Together, they balance ease of processing with mechanical robustness. PP is hydrophobic, counteracting PA6’s moisture sensitivity when paired, thereby stabilizing the hybrid assembly under humid conditions. It has lower modulus (~1.15 GPa) but superior impact resistance and ductility.

When the combined effect of PA6 and PP is considered, PA6 provides stiffness and adhesion, while PP contributes to energy absorption and crack-blunting. This duality enhances the crashworthiness of the aluminum tube system.

### 2.2. Specimens and Impact Tests

To absorb the impact energy of vehicles, the automotive industry uses steel passive protection bars (intrusion beams) in various dimensions. Inside the panels and doors, the diameters can be found between (30 and 40 mm) [25]. The hollow steel tube used in regular vehicles as a passive protection bar in the automotive industry is designed for impact absorption. Considering the sizes of steel tubes reported in the literature, we assume the outer diameter and wall thickness are about 30 mm and 1.5 mm, respectively. In the metal market around the Sakarya region, Türkiye, a standard Al tube with an outer diameter of 31 mm and a nominal wall thickness of 1 mm is selected. The length of the samples (L) is set to 270 mm to accommodate the pendulum impact test setup.

For this research, instead of a steel tube, various composite beam combinations are preferred as intrusion beams. The composite intrusion beams are composed of Al, PA6, and PP with different diameters. Given the high potential of polymer composite structures, the selected 31 mm outer diameter thin Al tube, supported by polymer materials as internal reinforcements, may increase the structure’s shock-absorbing capability. To understand the effects of internal reinforcements that absorb impact, we selected four specimen types for bending impact tests: an Al tube containing PA6 (constant thickness), a PP with two different thicknesses, and an Al tube without any reinforcements.

**Figure 2:**
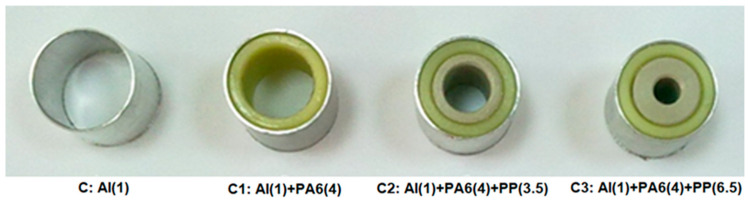
The specimens consist of aluminum tubes internally reinforced with polymer composites, with reinforcement thicknesses (mm) specified in parentheses.

For the inner reinforcement components, the outer and inner diameters of the polymeric specimens were machined from the supplied solid bars. Given the limitations of the machining process, a 4 mm thick, 29 mm outer diameter PA6 filler was manufactured for the first internal support of the hollow Al tube. Regarding the 21 mm outer diameter, PP specimens with 3.5 and 6.5 mm thick hollow sections were used inside the PA6 to serve as a second internal reinforcement of the Al tube due to the aforementioned limitations in PA6. Figure 2 shows the cutaway views of the polymer composite specimens, while Table 2 shows the details of the initially selected combinations of the polymer-reinforced composites.

**Figure 3:**
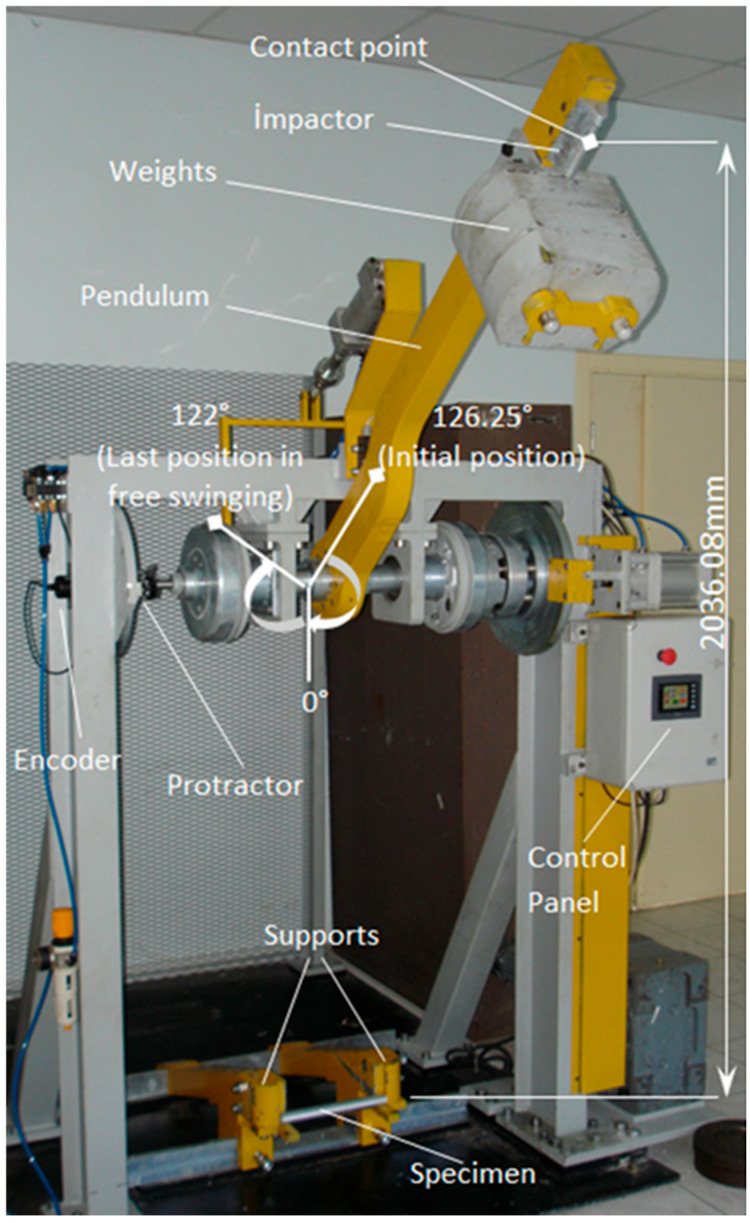
Bending impact pendulum test rig.

To investigate the impact resistance of the polymer-reinforced Al tubes, the bending impact tests were conducted with the pendulum test rig in the Mechanical Engineering Department of Sakarya University, as seen in Figure 3, to establish the design of the composite tube reinforced by the first filler, PA6, in the Al tube, and the second filler, PP, in the PA6 tube.

The V-shaped impactor (plunger) mass is 224 kg. The drop height of the pendulum for the initial position (126°) is 2036 mm, while the maximum drop height of the center of gravity (COG) is 1354.02 mm. However, the pendulum’s top speed at the specimen’s contact position is approximately 5000 mm.s^−1^, but we used the exact value of 5169 mm.s^−1^. When the swing angle of the pendulum is recorded employing a mechanical protractor, an encoder records the electronic data of the angle, too. The pendulum used a primary energy approach based on the COG heights, while the specimen used a swing system. The absorbed energy was calculated from the distance between the heights of the pendulum’s COG at the initial and the final positions. Thus, the absorbed energy required to deform the specimen was determined by the energy difference of the measured COG heights from the pendulum test rig (Figure 3).

To clarify the reasons for the improvement in the composite beams, we have used data gained from quasi-static three-point bending experiments by Eksi and Genel [26] on the 10 kN bending test machine at the same department of Sakarya University.

### 2.3. Numerical Analysis and Validation

To enhance bending impact testing of composite tubes across diverse scenarios, composite beam combinations were simulated in ANSYS/LSDYNA under dynamic bending impact conditions. Regarding the impact scenarios, the LSDYNA code is reliable and widely used for explicit dynamic (time-dependent) analyses in scientific studies [1,2,10,25,27,28]. Even though the LSDYNA code is an effective tool for engineers, validation of the simulation with experimental data is needed. By optimizing the explicit FEM data, we can approximate more than 90% of the test results, avoiding unnecessary, time-consuming, high-cost tests.

**Figure 4:**
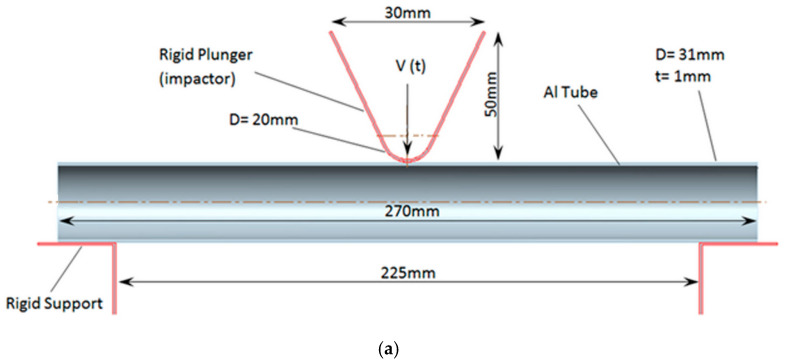

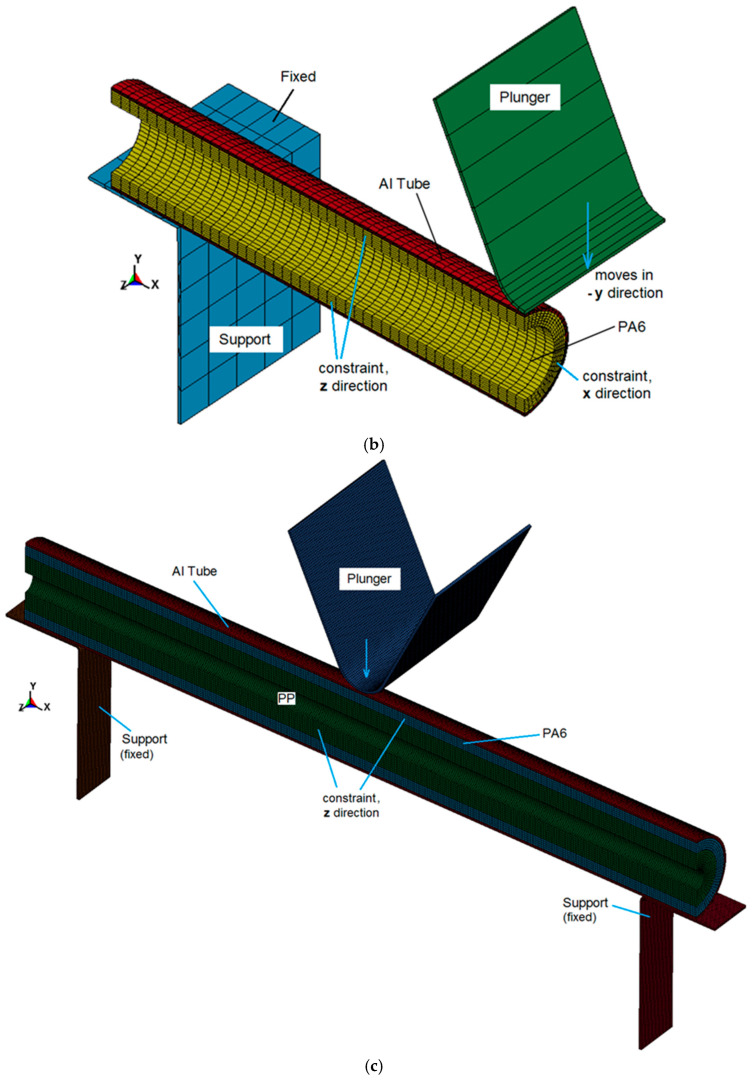
(**a**) The dimensions of the contact regions of the plunger, the hollow Al tube, and the supports. (**b**) The quarter finite element model of the composite beam: an Al tube (1 mm) reinforced by inner filler PA6 (4 mm). (**c**) The longitudinal half-finite element model of the Al tube reinforced by inner polymer fillers, PA6 (4 mm) and PP (6.5 mm).

To define the FE model, the constraints should be introduced first. For the FE model, the distance between supports is constant throughout all bending impact tests, and the span is 225 mm. The impactor (plunger) acts on the pendulum beam at the pendulum’s contact area, which has a diameter of 20 mm; other dimensions are shown in Figure 4a. To simplify the scenario, FE models were created at quarterly intervals. During the creation of the quarter models, the half tubes have sectioned surfaces constrained along the x-y symmetry plane (in the z direction). When reducing the model from half to quarter, the quarter FE models also exhibit sectioned surfaces parallel to the y-z symmetry plane (in the x direction), as depicted in Figure 4b, where the plunger acted. Because the quarter FE models were constrained along the y-z symmetry plane in the region where the plunger acted, we were unable to describe the details of crack growth and fracture mechanisms at the center of the tube. Thus, to investigate the central deformation region and bending-induced failure, longitudinally half FE models were created for broken specimens, with half tubes having sectioned surfaces constrained along the x-y symmetry plane (in the z direction), as we did before (Figure 4c). For both quarter and half models, the supports were fixed. At the same time, the plunger can move in the negative y direction.

Considering the contacts between the polymeric PA6 first inner reinforcement and the Al tube (specimen C1), they could pose a greater potential to cause various problems than the non-reinforced Al tube. Thus, a simpler description of the quarter-polymer composite FEM of a hollow Al tube reinforced with PA6 filler, with a 4 mm specified wall thickness (Figure 4b), was the C1 specimen, a prior quarter-polymer numerical model for initial validation. If we validate the experiments with simulations for the prior C1 specimen, using FEM optimization, the bending impact of the hollow Al tube is easier to assess.

Although the study’s initial methodology was to design the entire FE model for the composite specimens, simulating the entire tube proved time-consuming and CPU-intensive; thus, we created quarter and half models. Subsequently, the results for all experimental specimens were cross-checked against quarter models for non-broken polymer-reinforced Al composites and against longitudinal half models for broken polymer-reinforced Al composites. Since the C1 specimen did not break during the bending impact tests, a quarter simulation model was used, and the test results were identical to those of the initial validation model for explicit dynamics bending impact simulations. Using the quarter C1 simulation model, it was clearly determined that the quarter model’s impact behavior results in a shorter processing time than the half model.

**Figure 5:**
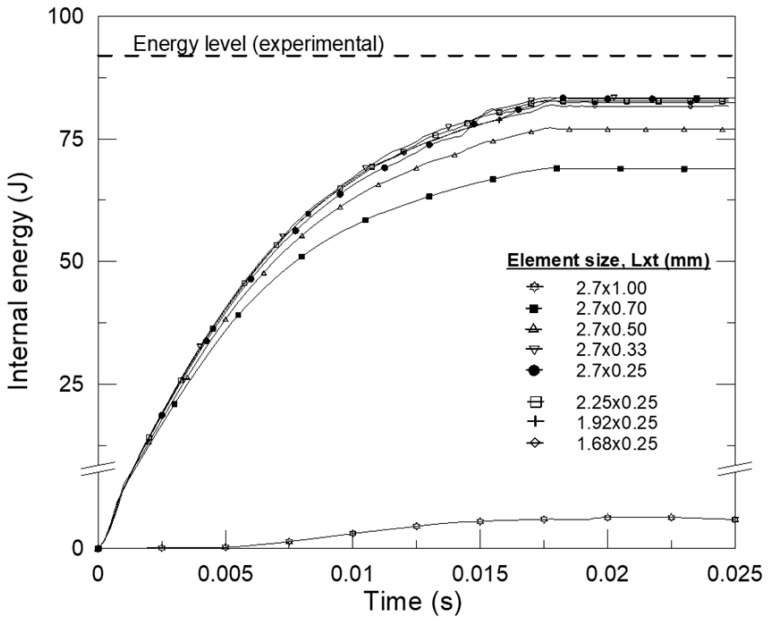
Internal energy-time history for various element sizes. (L: length, t: thickness).

In explicit FEM simulations, the mesh type and size are major factors in developing a more realistic model of impact-induced deformations leading to failure. For the simulations, hexahedral (solid 164) elements were preferred. For better convergence, the mesh sizes of the simulation models should be determined through mesh sensitivity analyses. To determine a convenient mesh size during impact, energy-absorbing levels were calculated for the entire specimen in all models; energies were multiplied by 4 for quarter models and by 2 for half models. Figure 5 represents the internal bending impact energy–time curves from the experiment and the quarter FE models in the various element sizes of the whole model of hollow Al specimens.

As shown in the figure, the simulation results are closer to the experimental results when the applied element size is preferred. Thus, the mesh element size was set to 2.7 × 0.33 mm for the quarter FE models, which was found to be reliable for the simulations and fast enough for the entire process duration, based on the mesh sensitivity analysis. To capture the details of crack growth in the broken composites, the mesh element size was reduced to 0.5 × 0.75 mm for the half models.

When the entire deformation procedure for bending impact is investigated, large deformations will occur throughout the entire specimen. The plastic kinematic material model with a hardening parameter (0.17) was used to represent the Al tube, and the failure strain option was used to characterize deformations more precisely in ANSYS/LSDYNA.

The pendulum’s speed was measured at 5169 mm/s during contact between the specimens and the V-shaped plunger, as in the experiments, while the plunger could move in the negative y direction. However, the total simulation period (or the contact duration) was approximately 25 milliseconds. To improve the precision of the computer simulations, the static and dynamic friction coefficients were determined from friction measurement tests to be 0.2 and 0.15, respectively. For the quarter FE models, the “automatic single-surface” method was used for contact between all materials, including self-contact, to prevent interlocking and self-recognition malfunctions. In contrast, the “surface-to-surface” contact type was used for the contact between the aluminum wall and the polymeric reinforcements. In the half FE models, the “eroding single-surface” and “eroding surface to surface” contact types were used to model crack initiation and fracture behaviors. The experimental load steps are the same for the simulations, and the experimentally identified pendulum energy was applied as an initial velocity to the V-shaped impactor. During the simulations, the number of output steps for the results file’s output interval and time history output interval was set to 100.

The validation model for the impact simulation should produce results identical to those of the experiment during comparison. As previously noted, a C1 specimen with a 4 mm thick PA6-filled 1 mm thick Al hollow tube was used to validate the initial finite element model. Numerical studies for the other beam combinations are summarized in Table 2. When the validation model was developed, the absorbed amount of bending impact energy was evaluated from both simulation and experimental results. The spring-back deflection of the composite beam was ignored to maintain the simulation’s focus.

When defining the validation model’s accuracy, the first simulation should be investigated in detail to examine the stress distribution along the specimen and the proportion of bending impact energy absorbed. Von Mises (v-m) stresses the importance of understanding stress localizations and the general stress distribution. Additionally, the C1 specimen’s deformed shape and characteristics were compared with the experimental results, which have similar plastic bending attributes and will be described later.

**Figure 6:**
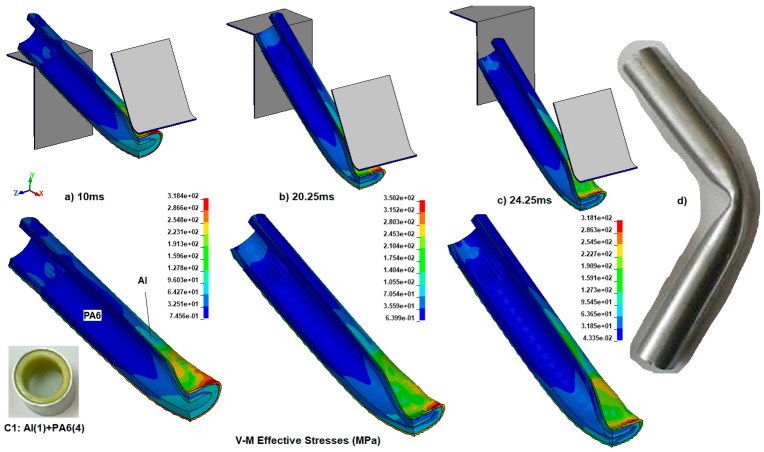
The Von Mises stress (MPa) contours at the time of (**a**) 10 ms, (**b**) 20.25 ms, (**c**) 24.25 ms, and (**d**) the experimental result for the specimen C1.

In Figure 6, the Von Mises stress contours (MPa) are shown at 10, 20.25, and 24.25 ms for the C1 simulation model.

Between the steps of Figure 6a–c, the stress concentrates in the vicinity of the loading point and the contact region of the supports and the Al tube. When the figures are examined, it is observed that folding begins with an increase in the impactor’s displacement and with stress localization at the corner of the tube section. The maximum stress occurs at 20.25 ms, about 350.2 MPa, as seen in Figure 6b. In the last stage at 24.25 ms, the composite specimen continued to move between the constant support spacing. Thus, after the first peak reaction during entry between the supports, the v-m stress decreased to 318.1 MPa as the specimen continued to move between the supports. After severe deformation in the middle region of the specimen, in which the upper and lower portions of the wall of PA6 contact each other (Figure 6c), a hinge mechanism finally occurred without any fracture or breaking. The hinge mechanism also occurred in the simulation model. The experimental result for specimen C1 is depicted in Figure 6d for validation of the simulation model.

**Figure 7:**
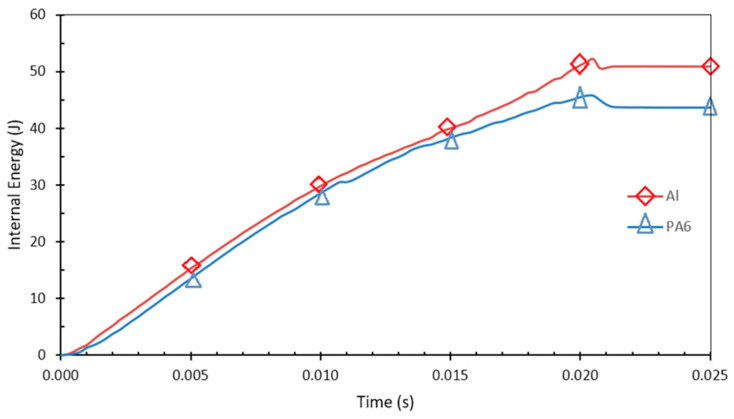
The absorbed impact energy versus time for the C1 specimen: Al tube and the first inner reinforcement of PA6.

To understand each material’s reaction during impact, Figure 7 represents the absorbed internal bending impact energy versus time for the Al tube and PA6 materials separately.

As for the total absorbed energy from the explicit FE simulations, it was calculated to be 378.276 J, the summation of the energies of the hollow Al tube with PA6 reinforcement multiplied by four, considering the quarter model. The three experimental results for the energy-absorbing capability of the C1 specimen are 389.31 J, 383.22 J, and 375.85 J. Finally, specimen C1 absorbed a mean bending impact energy of 382.8 J experimentally. From these results, it is possible to say that the finite element model and the simulation methodology used are appropriate, and the convergence error lies between 0.65% and 2.91%.

Hence, the finite element model and procedures for the C1 specimen were validated and adopted for the C, C2, and C3 simulation models as a convenient approximation.

Even for the uncomplicated material model, which is also preferred in this study, we need to focus on the final stage: does the selected FEM model validate the structure’s deformation and represent the impact effects? Although we deal with the details of these types of impact deformations, given the large deformations rather than focusing on small changes, we need to account for deformation characteristics and reduce shock effects by measuring the absorbed kinetic energy of the impact. Eventually, the final deformed shapes are validated against absorbed energies, and the simulation results closely resemble the shapes of similar deformed structures, with more than 90% convergence. Thus, the preferred material model worked properly enough to represent the deformation behavior.

## 3. Results and Discussion

### 3.1. Experimental Results

**Figure 8:**
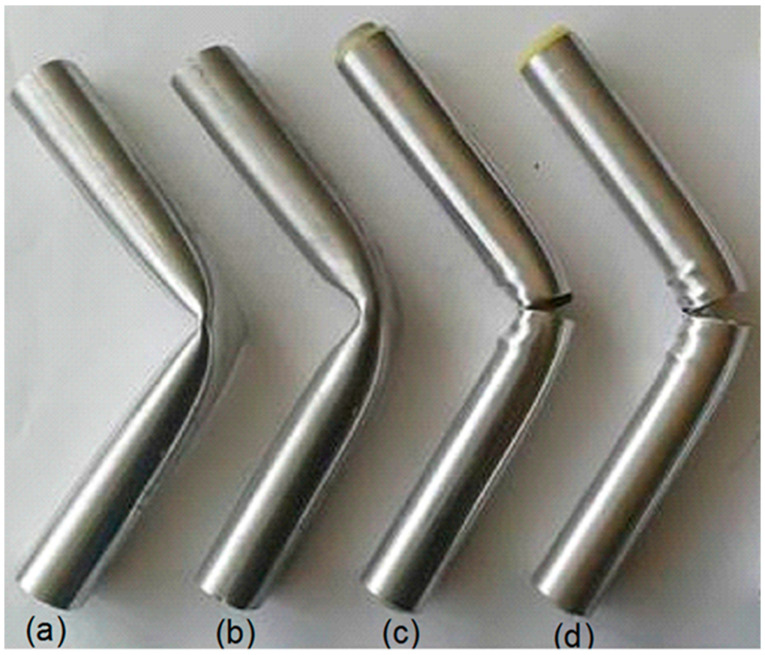
The specimens after the impact test. (**a**) C, (**b**) C1, (**c**) C2, and (**d**) C3.

Having performed the bending impact experiments allows us to comprehend the tendency of the supported composite beams and the hollow tubes. After the impact test, Figure 8 shows the representative view of the deformed specimens.

Plastic hinge formation can be seen in the unreinforced Al tube (Figure 8a), and the local deformation in the middle of the reinforced specimens decreases in the plain tube, as in some studies. Additionally, we observe a similar, but less pronounced, hinge formation due to the inner reinforcement of the PA6-filled Al tube (Figure 8b). The cross-sectional moment of inertia and the buckling behavior of the tube’s upper portion affect the bending performance, as reported in various studies [17,18,29]. The wrinkling around the contact region of the impactor increases with the increment of the inner reinforcement and the bending radius. This behavior reflects the bending resistance of the composite beams from the internal reinforcements. Thus, the composite specimens’ bending impact energy-absorbing capability is enhanced with support inside, while the bending radius (curvature) increases as expected. The wall of the Al tube tends to tear as the reinforcement efficiency of the materials increases, where PP was used inside 4 mm thick PA6 for the specimens C2 (3.5 mm thick PP) and C3 (6.5 mm thick PP) (Figure 8c,d).

The results of experiments and simulations of composite beams are presented in Table 3, along with the convergence ranges between them, and the “absorbed energy/mass” as defined by Specific Energy Absorption (SEA) values is also provided. The absorbed energy values in the table for the experiments are for the entire composite beams, measured from the test rig and calculated. Based on the simulations, we have two buckled hinge specimens (C and C1) and two broken specimens (C2 and C3). After validating the simulation results against experiments on the C1 specimen, the C specimen (a hollow Al tube) was also found not to break, allowing us to simulate it using a quarter model. Moreover, the PP-reinforced C2 and C3 specimens were broken, which cannot be modeled using a quarter model, as we mentioned before. To observe the onset of the crack and its growth till failure, we need to use a half-simulation model. For quarter models, to calculate absorbed energy, we will sum the composite components’ behavior and multiply by 4; for half models, we will multiply by 2.

When the test results are reviewed, inner reinforcement with polymeric materials significantly improves the Al tube’s bending impact energy-absorbing capability, as evidenced by the C1 specimen, by about 4.3 times, as indicated by the average experimental results. Unfortunately, the 3.5 mm- and 6.5 mm thick PA6-reinforced C2 and C3 specimens broke. Although we supported the composite tube with a polymeric reinforcement of PP inside PA6, the composite tubes failed in bending, rather than showing an increase in bending failure with increasing cross-sectional moment of inertia. During the experiments, there was no high-speed camera to record crack growth, so we were unable to see the details of the failure. Because we have filled tubular structures, if the polymeric supports crack and fracture before the outer aluminum tube, there is no way to record the internal deformation, even with a high-speed camera. The explicit dynamic simulations give us a chance to clarify the polymeric support deformations before the outer aluminum tube’s fracture. If we measure the energy change and observe the final deformation, we compare the energy-absorbing values of experiments and simulations and converge on more than 90–95 percent that will satisfy the experiments. Thus, the failure mechanism will be analyzed using explicit finite element simulations later.

**Figure 9:**
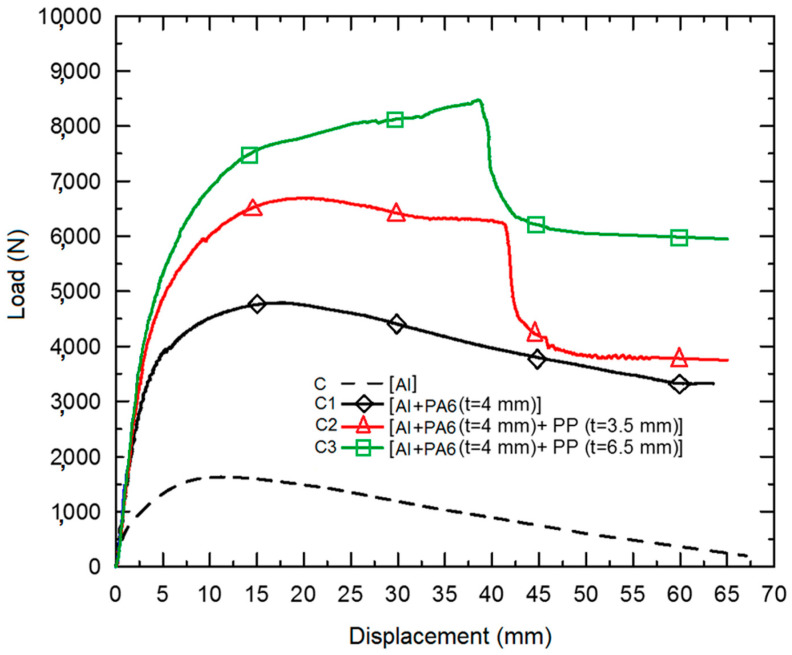
The quasi-static load-displacement curves of the specimens during bending.

To understand the failure mechanism, we first wanted to examine the quasi-static loading behaviors of the materials. Eksi and Genel [26], who are also from the same department, were previously studied using the same specimens but in quasi-static bending tests. According to their study, the load-displacement curves of the specimens shown in Figure 9 emphasize the reactions of C2 and C3 during quasi-static bending. During these tests, they found that the inner reinforcements increased the load-carrying capacity and the beam’s toughness, as evidenced by higher bending loads and buckling displacements. In addition, the inner reinforcement material hinders local buckling; thus, the buckling resistance increases accordingly. They have also stated that after the peak buckling deformations, the load-carrying capability of the C2 and C3 specimens decreased suddenly, caused by the tearing of the tube’s bottom location.

This region was subjected to severe tensile stress due to the strain limitation in the radial direction of the inner reinforcement materials. Considering the failure displacements of C2 and C3 specimens, the failure formation is directly related to the stiffness of the reinforcements.

A similar behavior was also observed in the bending impact tests. In addition, increasing the inner reinforcements and thicknesses did not have a positive effect. It has a disadvantage, particularly in high bending displacements, when the continuity of the deformation is considered. For the specific composite beam, the general form of the bending stiffness (BS) can be calculated as follows:

(1) $$BS=\sum_{i}^{n}E_{i}I_{i},$$

where *n* is the number of walls, E is the elasticity modulus in MPa, and I is the inertia moment in mm^4^.

**Figure 10:**
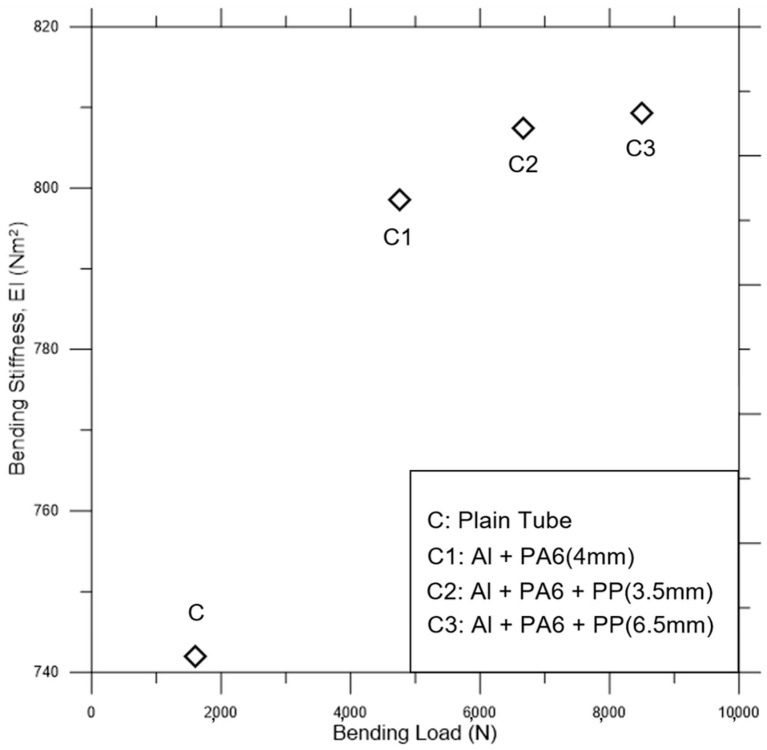
Quasi-static bending load versus bending stiffness of the specimens.

Figure 10 shows the quasi-static bending load versus bending stiffness for the specimens. It is possible to say that the bending performance of the composite is significantly increased depending on the reinforcement material’s contribution to the beam’s cross-sectional stiffness. Although there is not much difference between the bending stiffness of the composite beams, the bending load is considerably increased by the reinforcement (see C1–C3 in Figure 10). Although the bending stiffness increases for the PP reinforced C2 and C3 specimens, it is not enough to avoid bending failure under extra-large loading conditions.

Depicting “the hindering of local buckling” on the bending improvement of the beams reveals that the bending stiffness is a significant factor for the hollow tube. Preventing local buckling is a primary effect of composite beams; therefore, the effects mentioned above are the main factors that improve composite performance [4,29]. Previous studies [30,31] found that load-carrying capacity was associated with local buckling. Until the displacement at which the load reached its maximum value, the load was mainly carried by the bottom portion of the tube’s cross-section.

To clarify, the effect of bending stiffness is that when it increases under quasi-static loading, its capability to hold static forces also increases. However, for explicit dynamic deformations, it would not work at the same performance. In this study, we will examine the details of deformations during impact, which sometimes increase breakability and amplify failure mechanisms in hollow fillers.

Considering the quasi-static bending test results, the failure mechanism explains the composite beam’s basic impact behavior. After the quasi-static bending experiment curves in Figure 9, the bending stiffness data in Figure 10 continued to show the quasi-static behavior of the specimens. Although the impact deformations differ from the provided static test results to understand the fundamental static approaches, we need to demonstrate how tubular structures bend statically to provide a starting point. However, for this study, we will focus on impact scenarios that share similar response measures at only some levels for static tests. The quasi-static bending experiments were not simulated, as the test results are adequate to compensate for the explicit dynamics simulation data on bending impacts.

During the impact tests, a large load was applied to the composite tubes by the pendulum, which pushes the tubes between the supports and continues pushing them to the ends of the supports. After extra-large deformations, the tubes bend, and hinges form with no return to the initial state in any elastic region. Thus, the springback after this deformation has no effect on all of the materials used.

### 3.2. Explicit Finite Element Analysis Results

**Figure 11:**
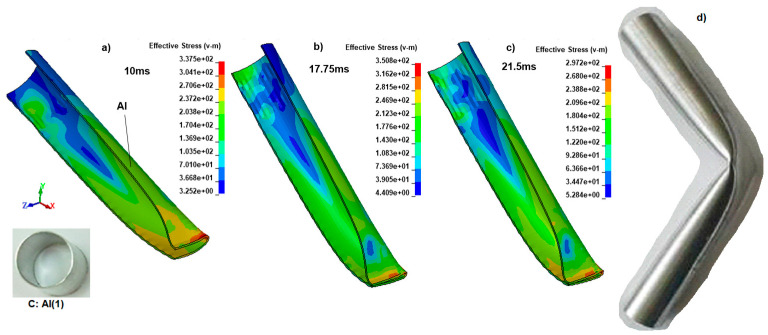
The Von Mises stress (MPa) distributions at (**a**) 10 ms, (**b**) 17.75 ms, (**c**) 21.5 ms, and (**d**) the experimental result for the specimen C.

After the experiments, the deformation process, fracture initiation, crack growth, and failure (breakage) could not be determined precisely. To clarify the deformation and failure procedures of the specimens, time-dependent explicit dynamic simulations were run for all specimens using the LSDYNA explicit solver. After experimental validation of a not broken prior quarter finite element model of specimen C1, the results were shown in Figure 6a–c. To continue with the not broken specimen C, which is a hollow, plain Al tube, the methodology of the C1 specimen was also applied to this FE model. Figure 11 represents the bending impact simulation steps for the other specimen, C.

With increased support from the polymeric inner reinforcements, the bending deformation behavior begins to change. That is why we have selected the critical deformation steps to correctly determine each specimen’s behavior, unless we need to apply the same time steps.

Figure 11a–c indicates the general bending behavior of the hollow Al tube (C) for the 10 ms, 17.75 ms, and 21.5 ms. Local plastic deformation increased with increasing displacement of the V-shaped impactor at the corner of the tube section in the plastic hinge region at 10 ms, as shown in Figure 11a. The second stage at 17.75 ms depicts the specimen just before the supports (Figure 11b). Because the Al tube resisted entering between the supports, the reactions increased, and the Von Mises stress reached a maximum of 350.8 MPa. In the last stage, the tube is between the supports for a while after severe deformation in the middle part of the specimen, resulting in contact between the upper and lower portions of the tube, with one surface folding against the other (Figure 11c). It is clear that this region is under the maximum stress (297 MPa) and is critical to failure. Between 17.75 ms and 21.5 ms, a hinge mechanism occurred without fracture because there is no reinforcement within the Al, and the tube can buckle easily in the plastic zone. The average absorbed impact energy calculated from the hollow Al tube experiments was approximately 85 J. The deformed experimental specimen of C is shown in Figure 11d, while the simulation model captured the hinged deformation observed in the experiment.

Nevertheless, the general stress at other locations in the Al tube was about 150 MPa. Additionally, using the quarter model, which held the longitudinal center of the specimens, we were unable to detect wrinkling on the top surface of the Al tube as an insufficiency. However, approximately 98% validation was achieved using simulation results from specimen C1, which served as a prior validation model, and from specimen C (a hollow Al tube), which was compared with experimental results.

**Figure 12:**
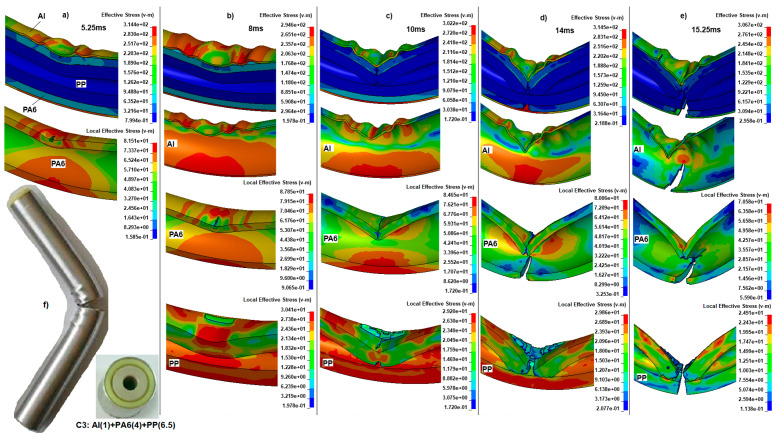
The Von Mises stresses (MPa) in local (polymeric materials) and global (entire specimen) crack initiation and growth until failure: the steps at (**a**) 5.25 ms, (**b**) 8 ms, (**c**) 10 ms, (**d**) 14 ms, (**e**) 15.25 ms, and (**f**) experimental result for the specimen C3.

After the experimental validation of the specimens C and C1, which used not broken quarter finite element models, we created half-finite element models for the broken specimens C2 and C3. To ensure that the bending impact behavior and failure of the specimens C2 and C3 were similar, the deformation steps of the thicker PP-reinforced Al and PA6 specimen C3 will be described. Using the Von Mises stress definitions in the local (polymeric materials) and global (entire specimen) domains, we will be able to characterize crack initiation, growth, failure, and rupture for the specimen C3 in Figure 12a–e.

Figure 12a–e shows the deformation behavior, crack initiation, and growth in each layer of specimen C3. The deformation steps differ from those of the others due to the changing thicknesses of the polymeric reinforcements, leading to different reactions. After the lower portion of the wall was stretched during the initial stage, wrinkling occurred at the upper portion of the wall, opposite the quarter model at 5.25 ms (Figure 12a). However, we emphasize that the half model can detect wrinkling in the Al tube. Also, a crack initiated at the top center of the first inner filler PA6 as the displacement increased because the v-m stress exceeded the plastic failure stress, with a local stress of 81.51 MPa. In contrast, the entire composite structure’s maximum stress was 314.4 MPa at some locations. The PP material’s stress was so small that it did not affect the structure. Although the maximum stress was that high due to the inner fillers, the Al tube did not deform much.

While stress increased at 8 ms, the top surface of the Al tube wrinkled further, reducing stress due to redistribution to the stretched section to 294.6 MPa at the bottom surface (Figure 12b). During this phase, the crack was growing under a v-m stress of 87.85 MPa from the top center of PA6 toward the back. At the same time, the PP was compressed (30.41 MPa) on the backside of the top surface.

At the 10 ms stage, the total stress began to increase again to 302.2 MPa as it tried to form a hinge. Still, the inner reinforcements prevented the Al tube from deforming, as shown in Figure 12c. As a result, the PA6 top surface was completely fractured, and the stress decreased to 84.65 MPa after failure. The compressed top of the central back of PP was further crushed, and two cracks from the top part of the tube’s back, bottom, and top grew and reached the center with a v-m stress of 29.2 MPa, more than the rupture point of PP.

In the next phase at 14 ms, the top center of the Al tube buckled sharply and fractured from the center to the back under v-m stress of 314.5 MPa, depicted in Figure 12d. Although the top surface had failed, the bottom portion of the Al tube continued to resist. We expected the Al tube to fail before the polymeric reinforcements, but at 10 ms, due to the stress concentration at the top (84.65 MPa), the PA6 at the bottom (80 MPa) broke, splitting into two pieces. In the meantime, the top center zone of PP was severely deformed, with the local v-m stress being 29.86 MPa above the plastic failure region.

The final failure step of the C3 specimen occurred at about 15.25 ms. Immediately after, the Al tube reached a maximum v-m stress concentration of 314.5 MPa at 14 ms (Figure 12e). The bottom part suddenly fractured laterally, accounting for more than half of the tube at 15.25 ms. The step before, the PA6 was broken, and due to relaxation, the stress decreased to 70.58 MPa. The similar failure behavior of the Al tube was observed in PP, with a sudden breakage of the remaining, unbroken part, splitting it into two pieces. The experimental result of the specimen C3 is given in Figure 12f. When we compare the experiment with the simulation, we found that increasing inner reinforcements causes the hinge mechanism to disappear and fractures due to the increasing bending radius. Based on the explained bending failure steps for C3, the mean absorbed impact energy was 367 J.

Considering the splitting failure deformation of the specimen C3, it was not applicable as a passive protection bar. However, the simulation results validate the experimental data with approximately 91% correctness.

When the specimen C2 is compared with C3, C2 shows similar deformation characteristics. While the thickness of PA6 for the specimen C2 was the same as that of C3, the thickness of PP was smaller, that is, about 3.5 mm. Because of its smaller thickness, the C2 will have a smaller cross-sectional moment of inertia, resulting in lower stiffness and bending resistance.

The lower thickness of PP led to earlier crack initiation at 5 ms, with PA6 at the same phase. At 8 ms, we first observed deformation of PP in specimen C3. In addition, the top center of the PA6 was fractured at 10 ms for C3, but for C2, a similar deformation was at 6 ms. Thus, we can determine that the analogous instant and total breakage failure of PA6 occurred during 14 ms, considering C3. However, this time, the lower thickness of the PP, because of a little more bending before the top and bottom parts of the PP came together and touched, delayed the breakage of the Al tube. The delay mechanism was extended, and total failure continued until the last splitting situation, specimen C2 at 20 ms, while C3 split at 15.25 ms. As we saw in C3, the specimen C2 also lacked a hinge due to the increased bending radius, despite a 3.5 mm thick PP layer. However, because of its lower cross-sectional area, the average absorbed impact energy of C2 is 183 J, almost half that of C3.

Furthermore, one of the most important factors is the Specific Energy Absorption (SEA), which represents the absorbed energy per mass of the specimens (see Table 3). When the specimens C2 (SEA: 0.885 kJ.kg^−1^) and C3 (SEA: 1.563 kJ.kg^−1^) were already torn apart, we could not use them for any structural applications under extreme loading conditions. However, for the hollow Al tube (specimen C), SEA is 1.245 kJ.kg^−1^, and for the Al tube internally reinforced with 4 mm thick PA6 (specimen C1), SEA is 2.331 kJ.kg^−1^, which clarifies the increment of the developed composite combination.

To determine whether we can offer an unbroken polymer-reinforced composite tube combination, we have only simulated using the validated FE modeling method with two additional PP thicknesses, 8 mm and 9 mm, and an additional filled PP model inside a constant 4 mm thickness of PA6, as we have used from the beginning.

For the 8 mm thick PP inside the 4 mm thick PA6 is specimen C4, and the 9 mm thick PP used inside the PA6 is specimen C5, while the full PP used inside the PA6 is specimen C6. After the bending impact simulations, specimens C4 and C5 continued to fail by splitting.

**Figure 13:**
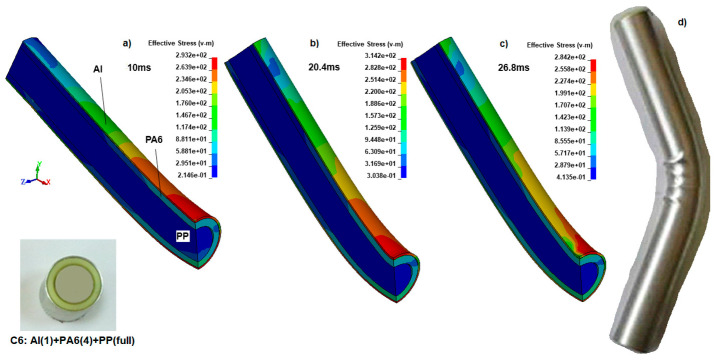
The Von Mises stress (MPa) distributions at (**a**) 10 ms, (**b**) 20.4 ms, (**c**) 26.8 ms, and (**d**) experimental result for the specimen C6.

In the end, filled PP inside PA6 (specimen C6) was not broken (Figure 13). From the simulation results, we found that when the PP inner reinforcement has a hole, it leads to bending failure. This means that for the 31 mm outer diameter Al tube with 1 mm thickness, it has not reached the critical cross-sectional moment of inertia to avoid bending failure. When the PP has no gap inside, the inner reinforcements support each other, forming a protective combination against the bending impact under extreme loading.

During the simulation of specimen C6 in Figure 13a–c, the causes of the hourglass parameter adjustments and the shape of the FE model were revealed. Initially, the bending compression region’s Von Mises stress was about 293.2 MPa at 10 ms, slightly lower than that of sample C3 (302.2 MPa), in which the PP is 6.5 mm thick. When the pressure was increased, considering the inner reinforcement, there was no buckling in the radial space, unlike in hollow tubes. Instead of buckling or localizing similar to the hollow ones, the entire structure’s radius increased; that is why this effect reduces local stress values. The ends of the C6 polymer-reinforced composite tube reached 20.4 ms between supports, whereas the same behavior in the broken C3 was 14 ms, and the unbroken C1 was 20.25 and close to C6, as expected. Also, the distance between the supports was the same, and the volume of reinforcement material was greater than that of all other samples (C-C5). However, stress is higher than for specimen C2 and is close to that of C3, while this sample is close to the buckling stage but has not buckled.

To evaluate the final developments of the specimens after C3, the experimental results are compared with the explicit FE simulation results, and the convergence rate error is shown in Table 4 along with the Specific Energy-Absorbing (SEA) capability.

**Figure 14:**
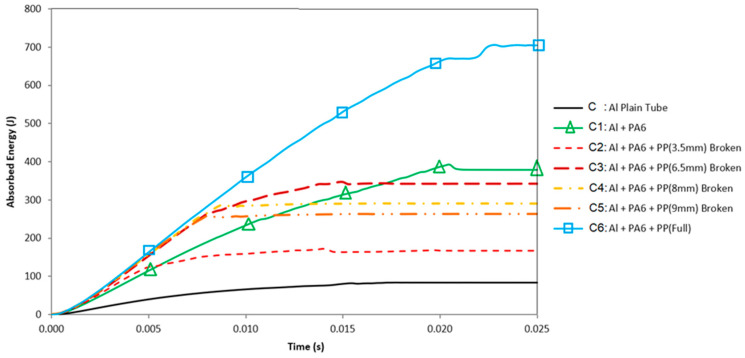
Absorbed energy versus time for all composite specimens, together with the unreinforced Al tube.

For effectiveness, we need to examine the bending impact-absorbing behavior in the study, from a hollow Al tube (specimen C) to a fully polymer-reinforced composite tube (specimen C6). The details of the bending impact energy-absorbing distribution for all specimens during explicit FE simulations, as explained so far, are shown in Figure 14.

For the reinforced specimens (C1–C3), there are unavoidable sudden fluctuations in the curves of absorbed impact energy at some locations (Figure 14). The oscillations arose from friction at the supports’ corners and the scraping effect of the contacting surfaces. These curve fluctuations increased with the addition of inner reinforcements due to the augmented reaction force. With the rise in the reaction forces, indentation occurs from the corners of the supports towards the tube’s surface. The indentation magnitude increased with the reinforcement effect, leading to fluctuations in the curve.

When the curve of specimen C1 in Figure 14 is considered, there was a significant increment in the impact energy absorption, which was directly related to the existence of PA6. At 20.25 ms, the specimen entered the span (between the supports) at the cornered supports, and friction held the sample in place. When the tube passed between the corners, it relieved and evaded the scraping effect of the corners, resulting in a considerable zig-zag in the curves. Here, the absorbed energy level decreased slightly immediately. This energy level continued till the specimen exceeded the span. Because there is no failure for C1, after C6, C1 is the most convenient specimen to use.

For specimen C2, the application of a 3.5 mm thick PP reinforcement dramatically increased impact energy absorption compared to specimen C1 before 5 ms, where the crack initiation occurred in PA6 (Figure 14). The specimen movement was more rapid, and the relief period arrived sooner. Thus, the starting point of the relief period was less than that of the previous model. When the relief period started, the absorbed energy level decreased. Unfortunately, after 5 ms, cracks formed and grew in all components of the specimen, eventually breaking it. A zig-zag form in the curve was also observed for this specimen.

The absorbed impact energy of specimen C3 increased initially because of the thick inner reinforcements. Until the total failure of the specimen, because the inner reinforcements’ top and bottom parts compressed and absorbed energy, which continued to rise. However, the amount of absorbed energy was greater than that for C2, as we expected. However, for C4 and C5, although we increased the thickness of PP to 8 mm and 9 mm, the energy absorption capacities were lower than those of C3 due to early-phase sudden cracks. When we checked the specimens C2, C3, C4, and C5, the cross-sectional moments of inertia were higher than those of C1, the unbroken and the only PA6-reinforced specimen. However, the increase in moments of inertia continued, and the bending radius grew as well due to the more resistant fillers. This formation caused early fractures in the specimens C4 and C5.

Considering the vertical diameter of the cross-section, it is deduced that deformation is limited in the critical region associated with the reinforcement effect of the polymeric materials. Hence, these reinforcement materials also restrict the formation of plastic hinges. Some studies emphasize that local deformation and hinge formation play significant roles in bending behavior [8,17,18,28]. Based on the knowledge we have gained so far, we conducted experiments on specimen C6 and validated the simulations against it, as we mentioned before. When we checked the simulations, the cross-sectional moment of inertia was just strong enough to prevent the composite tube from breaking. At the final stage, specimen C6 has a mean experimental bending impact energy absorption of 749.41 J, 8.8 times more than the hollow Al tube’s impact-absorbing energy, which was 85.18 J. In addition, from the simulation comparison, the specimen C6 is 8.43 times more than the specimen C. If we look back at Table 3 and Table 4, we can see the error % between the experiments and simulations. Overall, the convergence percentage is about 95%, which is absolutely remarkable for this type of failure-induced explicit FE simulations. In addition, the improvement in Specific Energy Absorption (SEA) is approximately 3 kJ.kg^−1^, which is 2.4 times greater than the SEA of the hollow Al tube (1.245 kJ.kg^−1^; see Table 3).

**Figure 15:**
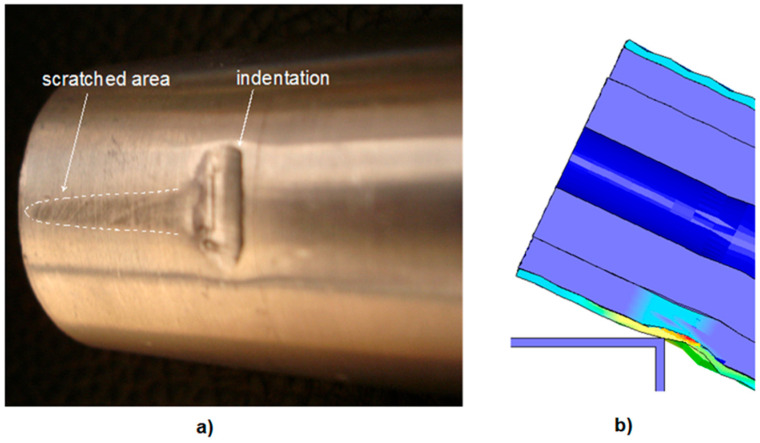
Formation of indentation and scratching: (**a**) appearance of specimen surface after the experiment and (**b**) during the test simulation.

During the deformation phases of all the specimens, due to the sharp-edged supports, we also observed annular indentations that resulted in scrapes on the specimens, as shown in Figure 15a,b. Focusing in detail on the contact region of the support’s edge and the specimen surface, the appearance of the specimen surface after the experiment shows a good agreement between the simulations and the test results.

Finally, the results of this study benefited from quasi-static deformation knowledge from earlier studies [26,30] and were used to validate experiments and simulations. Additionally, this research provides an opportunity to combine and enhance composite structural elements, which can be applied across various applications [32,33]. However, the enhanced polymer-reinforced tubular composite, C6, which we need for extreme bending impact loads, is made from the thinnest available materials on the market; accordingly, we are proposing this combination to the literature.

## 4. Conclusions

Given the study’s approach, the pendulum test rig was developed to simulate the bending impact conditions encountered by the structural components. The effects of the inner polymeric reinforcements on the bending impact resistance of an Al tube (6063-T6) are investigated using ANSYS/LSDYNA to ensure the simulations for the bending impact deformation of composite beams are reliable enough to absorb the impact energy.

The local buckling of the tube and its reduced resistance resulted in a significant reduction in its energy absorption capability. Experimental results of unreinforced beams indicate that buckling usually occurs in the hinged region while the ends of the beam rotate rigidly at a slight angle.

From the quasi-static bending test results, the failure mechanism is apparent, and the composite beams’ impact behavior is evident. Hence, the deformation phases of the impact and quasi-static bending processes are similar, except for the material behavior under high-speed loading conditions.

The application of inner reinforcement materials improves the bending impact energy-absorbing capability of hollow Al tubes. If the inner polymeric reinforcements have a hole, they are more likely to fracture under extreme bending conditions. The inner reinforcements, consisting of 4 mm thick PA6 and a 21 mm-diameter full PP, offer superior energy absorption, helping to hinder local buckling. It is possible to say that significant profits are achievable in structures under the threat of bending impact loading. The proposed composite structure C6 is promising, especially for the critical parts that serve as vehicle support members. The impact energy-absorbing level of the internal reinforcements with polymeric materials is increased by 8.8 times compared to the hollow Al tube used in the automotive industry under bending impact loads. For a better comparison, the Specific Energy Absorption (SEA) for the plain aluminum tube (C) is 1.245 kJ.kg^−1^. With the first inner polymeric filler in specimen C1, the SEA was 2.331 kJ.kg^−1^, and for the final improvement in C6, we obtained 2.985 kJ.kg^−1^. The final development of the SEA of the hollow aluminum tube is 2.4 times more with the inner polymeric reinforcements, considering the passive protection applications in the mostly automotive industry.

The simulation results are consistent with the experimental results, providing us a mean convergence rate of 95%. Investigated numerical and experimental comparisons indicate that the developed C1 and C6 composite tubes are reliable and safe as passive protective components. The performed explicit FE analysis demonstrates that local deformation plays a critical role in hinge formation, using a systematic approach during time-dependent deformation steps, especially during crack initiation and growth until failure. From the detailed examination of the polymer-reinforced composite specimen deformations, the inner reinforcement improves impact energy absorption by delaying local deformation and hinge formation, provided that no crack growth occurs during deformation. Also, the failure mechanism primarily involves regional deformation, wrinkling, and hinge formation.

## Figures and Tables

**Table 1:** The properties of the Al tube and the inner polymer reinforcement materials.

Mechanical Properties	Al 6063-T6 Tube	PA6 Reinforcement	PP Reinforcement
Young Modulus (E) (Pa)	69 × 10^9^	2.25 × 10^9^	1.15 × 10^9^
Mass Density (ρ) (kg.m^−3^)	2.75 × 10^3^	1.15 × 10^3^	0.9 × 10^3^
Poisson Ratio (ν)	0.33	0.4	0.4
Yield Stress (σy) (Pa)	235 × 10^6^	60.75 × 10^6^	28 × 10^6^
Tangent Modulus (Etan) (Pa)	218 × 10^6^	131.59 × 10^6^	8.647 × 10^6^
Hardening Parameter (*n*)	0.17	-	-

**Table 2:** The combinations and the dimensions of the specimens.

Specimen	Materials from Outside to Inside	Outside Diameter (mm)			Thickness (mm)		
		Al	PA6	PP	Al	PA6	PP
C	Al	31	-	-	1	-	-
C1	Al + PA6	31	29	-	1	4	-
C2	Al + PA6 + PP	31	29	21	1	4	3.5
C3	Al + PA6 + PP	31	29	21	1	4	6.5

**Table 3:** Results of the impact tests and the impact simulations of the C, C1, C2, and C3 specimens.

Sample (Thickness)	Impactor’s Mass (kg)	Sample’s Mass (kg)	Simulation’s Absorbed Energy (J)	Experiment’s Absorbed Energy (J)	Exper. vs. Sim. Energy, Error (%)	Mean SEA (kJ.kg^−1^)
C: Plain Al (1)	224	0.069	83.468	85.94	2.96	1.245
				86.76	3.94	
				82.84	0.76	
C1: Al (1) + PA6 (4)	224	0.167	378.276	389.31	2.91	2.331
				383.22	1.30	
				375.85	0.65	
C2: Al (1) + PA6 (4) + PP (3.5)	224	0.214	167.718 *	189.38 *	12.92	0.885
				177.12 *	5.60	
				181.44 *	8.18	
C3: Al (1) + PA6 (4) + PP (6.5)	224	0.239	342.182 *	373.62 *	9.18	1.563
				366.53 *	7.11	
				357.71 *	4.53	

* Broken, (SEA: Specific Energy Absorption).

**Table 4:** Results of the impact tests and the impact simulations of the C4, C5, and C6 specimens.

Sample (Thickness)	Impactor’s Mass (kg)	Sample’s Mass (kg)	Simulation’s Absorbed Energy (J)	Experiment’s Absorbed Energy (J)	Exper. vs. Sim. Energy, Error (%)	Mean SEA (kJ.kg^−1^)
C4: Al (1) + PA6 (4) + PP (8)	224	0.245	290.1 *	NA	NA	1.184
C5: Al (1) + PA6 (4) + PP (9)	224	0.248	268.854 *	NA	NA	1.084
C6: Al (1) + PA6 (4) + PP (Full)	224	0.251	704.064	760.89	8.07	**2.985**
				741.14	5.26	
				746.20	5.98	

* Broken, (NA: not applicable, SEA: Specific Energy Absorption).

## Data Availability

The original contributions presented in this study are included in the article. Further inquiries can be directed to the corresponding author.

## Abbreviations

The following abbreviations are used in this manuscript:

PA6	Cast Polyamide
PP	Polypropylene
FEA	Finite Element Analysis
FEM	Finite Element Method
SEA	Specific Energy Absorption
NA	Not Applicable
